# The Middle Fragment of *Helicobacter pylori* CagA Induces Actin Rearrangement and Triggers Its Own Uptake into Gastric Epithelial Cells

**DOI:** 10.3390/toxins9080237

**Published:** 2017-07-28

**Authors:** Abolghasem Tohidpour, Rebecca J. Gorrell, Anna Roujeinikova, Terry Kwok

**Affiliations:** 1 Infection and Immunity Program, Monash Biomedicine Discovery Institute, Department of Microbiology, Monash University, Clayton, Victoria 3800, Australia; a.tohidpour@gmail.com (A.T.); rebecca.gorrell@monash.edu (R.J.G.); 2 Infection and Immunity Program, Monash Biomedicine Discovery Institute, Department of Biochemistry and Molecular Biology, Monash University, Clayton, Victoria 3800, Australia

**Keywords:** endocytosis, macrospike protrusions, integrin, actin polymerization, phosphatidylserine

## Abstract

Cytotoxin-associated gene product A (CagA) is a major virulence factor secreted by *Helicobacter pylori*. CagA activity in the gastric epithelium is associated with higher risk of gastric cancer development. Bacterial type IV secretion system (T4SS)-mediated translocation of CagA into the cytosol of human epithelial cells occurs via a poorly understood mechanism that requires CagA interaction with the host membrane lipid phosphatidylserine (PS) and host cell receptor integrin α_5_β_1_. Here we have characterized the isolated recombinant middle fragment of CagA (CagA-M) that contains the positively-charged PS-binding region (aa 613–636) and a putative β_1_ integrin binding site, but lacks the EPIYA region, secretion signal peptide and the CagA multimerization motif. We show that CagA-M, when immobilized on latex beads, is capable of binding to, and triggering its own uptake into, gastric epithelial cells in the absence of infection with *cagA*-positive *H. pylori*. Using site-directed mutagenesis, fluorescent and electron microscopy, and highly-specific inhibitors, we demonstrate that the cell-binding and endocytosis-like internalization of CagA-M are dependent on (1) binding to PS; (2) β_1_ integrin activity; and (3) actin dynamics. Interaction of CagA-M with the host cells is accompanied by the development of long filopodia-like protrusions (macrospikes). This novel morphology is different from the hummingbird phenotype induced by the translocation of full-length CagA. The determinants within CagA-M and within the host that are important for endocytosis-like internalization into host cells are very similar to those observed for T4SS-mediated internalization of full-length CagA, suggesting that the latter may involve an endocytic pathway.

## 1. Introduction

*Helicobacter pylori* is a Gram-negative, microaerophilic bacterium that colonizes the human stomach [1]. *H. pylori* infects approximately half of the world’s population. Colonization of the stomach by *H. pylori* can lead to peptic ulcers, active and chronic gastritis, non-Hodgkin’s lymphoma, and distal gastric adenocarcinoma [2]. Gastric cancer is the third leading cause of cancer-related death worldwide, with almost 1 million new cases occurring in 2012 alone [3]. *H. pylori* virulence is in part determined by whether a strain expresses the product of the cytotoxin-associated gene A (CagA) [4,5]. CagA is the first identified bacterial oncoprotein [6]; infection with a CagA-positive *H. pylori* strain results in elevated motility of human gastric epithelial cells due to disruption of apical junctions [7] and adherens junctions [8]. In addition, CagA increases proliferation and transdifferentiation of gastric epithelial cells [9,10]. CagA-induced dysregulation of the β-catenin signaling pathway [9,10] plays a central role in these pathogenic processes, and is thought to underpin the increased gastric cancer risk observed with CagA-positive strains [11].

The *cagA* gene resides in a 40-kb genetic locus known as the *cag* pathogenicity island (*cag*PAI) [6], which also encodes the components of a type IV secretion system (T4SS). This complex secretion machinery delivers CagA into the cytosol of the host cell. The exact mechanism by which the T4SS mediates CagA translocation into the host cell is poorly understood, however, it requires a secretion signal peptide located at the C-terminus of CagA [12]. Once translocated into the host cell cytoplasm, host kinases phosphorylate tyrosine residues within the glutamate-proline-isoleucine-tyrosine-alanine (EPIYA) motifs located in the C-terminal region of CagA. CagA binds, in a phosphorylation-dependent or phosphorylation-independent manner, to a range of host proteins including Src homology 2 (SH2)-containing tyrosine phosphatase 2 (SHP-2) [13], growth factor receptor-bound protein 2 (Grb2) [14], C-terminal Src kinase (Crk) [15] and microtubule affinity-regulating kinase 2 (MARK2) [16], resulting in dysregulation of key cellular biochemical pathways, apoptosis, and the multi-step development of gastric cancer [13,17,18]. Consequently, during *H. pylori* infection, numerous cellular responses are triggered by translocated CagA, including rearrangements of the host actin cytoskeleton that leads to the development of aberrant morphological changes to the cell. The resulting “hummingbird” morphology is characterized by cell elongation and formation of spindle-like cellular protrusions that contain actin filaments [13,17,19,20]. 

CagA internalization by human epithelial cells requires interaction with the host membrane lipid phosphatidylserine (PS) [21]. Although PS normally resides in the host cell membrane inner leaflet, it can transiently appear in the outer leaflet at sites of *H. pylori* attachment. CagA is believed to exploit PS in both the outer and inner leaflets for host cell translocation, and subsequent CagA localization to the inner leaflet. CagA anchorage occurs via electrostatic interactions between a putative lipid-binding region located in a cluster of conserved positively-charged residues on the solvent-accessible face of a CagA α-helix, and the negatively-charged phosphate groups of PS and phosphoinositides [22]. 

In addition to the interaction with PS in the host cell membrane, CagA delivery into the host cell also requires *H. pylori* binding to the mammalian transmembrane receptor integrin α_5_β_1_ [23,24,25]. CagA, and the T4SS structural subunits CagY and CagL, interact with integrin subunit β_1_; these interactions play key roles in CagA translocation into the host cell [23,24,25]. Integrins are important for bidirectional signal transduction across the plasma membrane, linking cytoskeletal responses to the extracellular matrix [26,27]. Apart from *H. pylori*, numerous other bacterial pathogens have evolved to interact with integrins for attachment to, and entry into, host cells. Notably, the basic PS-binding patch and the putative β_1_-integrin binding site are located on the same side of the CagA molecule [22,28]. It is possible that their juxtaposition has evolved to enable the CagA middle region to interact simultaneously with the host cell membrane and the receptor integrin α_5_β_1_.

**Figure 1:**
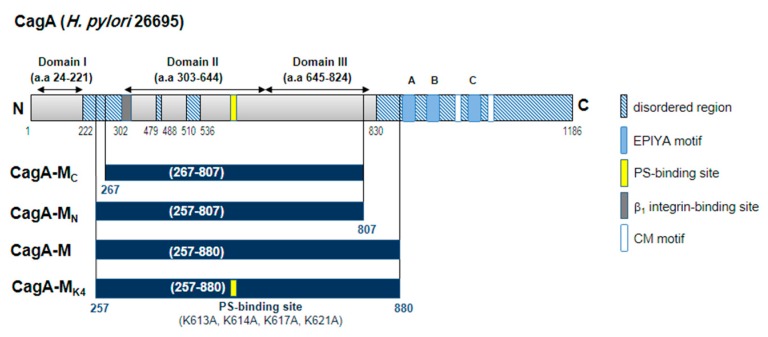
Schematics of full length CagA from *H. pylori* strain ATCC 26695 and the four CagA fragments used in this study, CagA-M, CagA-M_N_, CagA-M_C_, and CagA-M_K4_. Pale blue bars and capital letters A, B, and C show the location of the regions containing the EPIYA motifs A, B, and C. Hatched areas denote disordered regions. White bars indicate the CagA multimerization sites (CM motifs). Yellow and dark gray bars denote the PS-binding site and β_1_ integrin binding sites, respectively. The four lysine to alanine substitutions (K613A, K614A, K617A, K621A) generated to inactivate the PS-binding site on CagA-M_K4_ are also shown.

To better understand the molecular mechanisms underpinning CagA translocation across the host cell membrane, we investigated T4SS-independent interactions between gastric epithelial cells and isolated recombinant CagA fragments containing the positively-charged membrane tether (aa 613–636) [22,28] as well as a putative β_1_ integrin binding site (aa 303–368) [28] (Figure 1), but lacking the EPIYA region, secretion signal peptide and the CagA multimerization (CM) motif [29]. Correct folding of one such fragment, namely CagA-M, has been shown previously [22,30]. Recombinant CagA-M (residues 257–880) encompasses two middle domains (Domain II [aa 303–644] and Domain III [aa 645–824]), part of the linker between Domain II and Domain I (aa 24–221), and a flexible region 825–880. The proteolytically-stable core of CagA-M (fragment CagA-M_C_) has previously been delineated as residues 267–807, and contains Domain II, most of Domain III and part of the linker between Domain II and Domain I (Figure 1). We also generated an additional protein construct, CagA-M_N_ (residues 257–807), that has the same N-terminus as CagA-M and the same C-terminus as CagA-M_C_. In addition, we have produced a CagA-M variant, CagA-M_K4_ (257–880), that has lysine-to-alanine substitutions at positions 613, 614, 617, and 621 in the putative positively-charged lipid-binding region. These substitutions have been previously shown to significantly reduce the binding affinity of CagA fragments to mammalian phospholipid bilayers or artificial lipid mixtures [22]. 

Here, we present our analysis of T4SS-independent interactions of CagA-M, CagA-M_C_, CagA-M_N_, and CagA-M_K4_ with gastric epithelial cells, identify determinants within CagA and within the host that are important for such interactions, and discuss the implications of our findings for the mechanism of CagA internalization by the host cells.

## 2. Results

### 2.1. The Middle Fragment of CagA (CagA-M, aa 257–880) Alone Is Sufficient for Altering Host Cell Morphology

**Figure 2:**
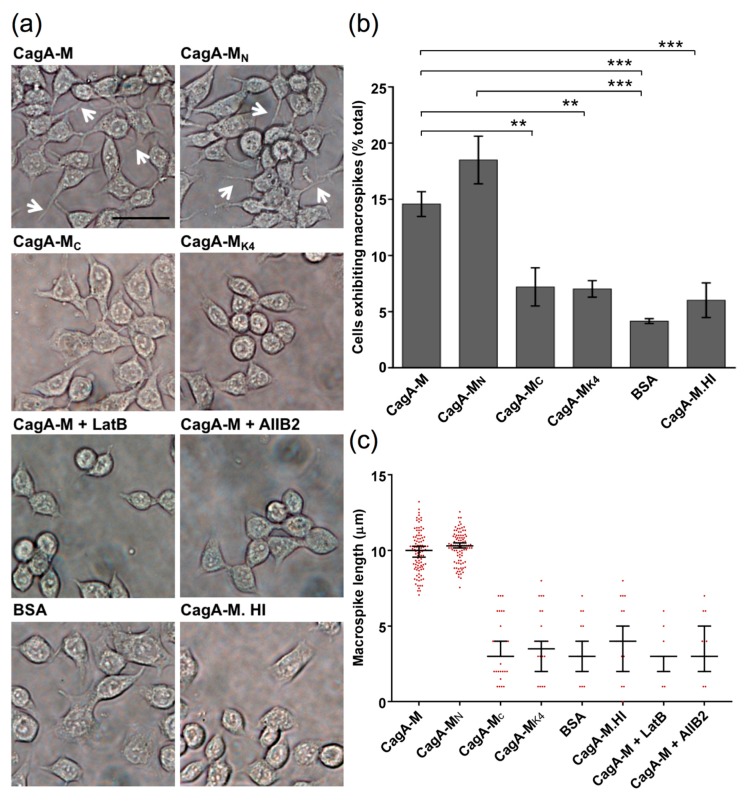
Induction of macrospike protrusions in human gastric epithelial (AGS) cells by exposure to the middle fragments of CagA for 24 h. (**a**) Phase contrast microscopy images of AGS cells treated with fragments CagA-M, CagA-M_N_, CagA-M_C_, and CagA-M_K4_. Also shown are images of AGS cells pre-treated with an actin inhibitor latrunculin B (LatB) prior to exposure to CagA-M (CagA-M + LatB) and AGS cells pre-treated with a β1 integrin blocking antibody (AIIB2) prior to exposure to CagA-M (CagA-M + AIIB2). Control samples included AGS cells exposed to bovine serum albumin (BSA) and heat inactivated CagA-M (CagA-M. HI). Macrospikes are indicated by white arrows. Scale bar, 20 µm. (**b**) Graph showing the percentage of AGS cells exhibiting macrospikes following treatment with CagA-M, CagA-M_N_, CagA-M_C_, CagA-M_K4_, BSA, or CagA-M. HI. All data are mean values ± standard error of the mean (s.e.m.) of three independent experiments (***, *p* < 0.001; **, *p* < 0.01). A total of 350 cells were counted per experiment. (**c**) Graph showing the average lengths of macrospikes in AGS cells post-treatment with CagA-M (with or without treatment with LatB or AIIB2), CagA-M_N_, CagA-M_C_, CagA-M_K4_, BSA, or CagA-M. HI. Each data point represents the average length of macropikes on an individual cell that exhibits at least one macrospike; a total of 100 cells were analyzed per sample per experiment. Representative data from three independent experiments are shown. Median values are indicated by long horizontal bars; short horizontal bars indicate 95% confidence intervals. The differences in the median macrospike length between CagA-M and other samples except CagA-M_N_ were statistically significant (*p* < 0.0001). Similarly, the differences in median macrospike length between CagA-M_N_ and the other samples except CagA-M were statistically significant (*p* < 0.0001). All other comparisons yielded statistically insignificant differences. CagA-M- and CagA-M_N_-treated AGS cells exhibited on average 2–4 macrospikes, whereas all of the other treatments resulted in only 0–1 macrospike per cell.

To first examine whether the middle fragment of CagA (CagA-M, aa 257–880) alone is capable of interacting with gastric epithelial cells, we incubated the human gastric adenocarcinoma cell line AGS with purified CagA-M (1 mg/mL) for 24 h and examined cell morphology using phase-contrast microscopy. CagA-M, but not bovine serum albumin (BSA) or heat-inactivated CagA-M, triggered long filopodia-like protrusions to form on AGS cells (Figure 2). We refer to these protrusions as macrospikes as they were longer and much thicker than typical filopodia, with an average length and diameter of approximately 10 µm (Figure 2c) and 1 µm, respectively. CagA-M triggered the formation of an average of 2–4 macrospikes per cell (Figure 2a), which conferred the cells a star-like morphology. The latter is distinct from the hummingbird phenotype (also known as elongation phenotype) induced upon *H. pylori* infection, which is characterized by tapered protrusions and a more elongated cell body [31]. We note that while the hummingbird phenotype requires *H. pylori* T4SS-dependent translocation of full-length CagA into the host cell cytoplasm, the development of the macrospike-containing star phenotype required only stimulation by CagA-M alone. 

### 2.2. β_1_ Integrin Activation Is Required for the Morphological Changes Induced by the Middle Fragment of CagA

**Figure 3:**
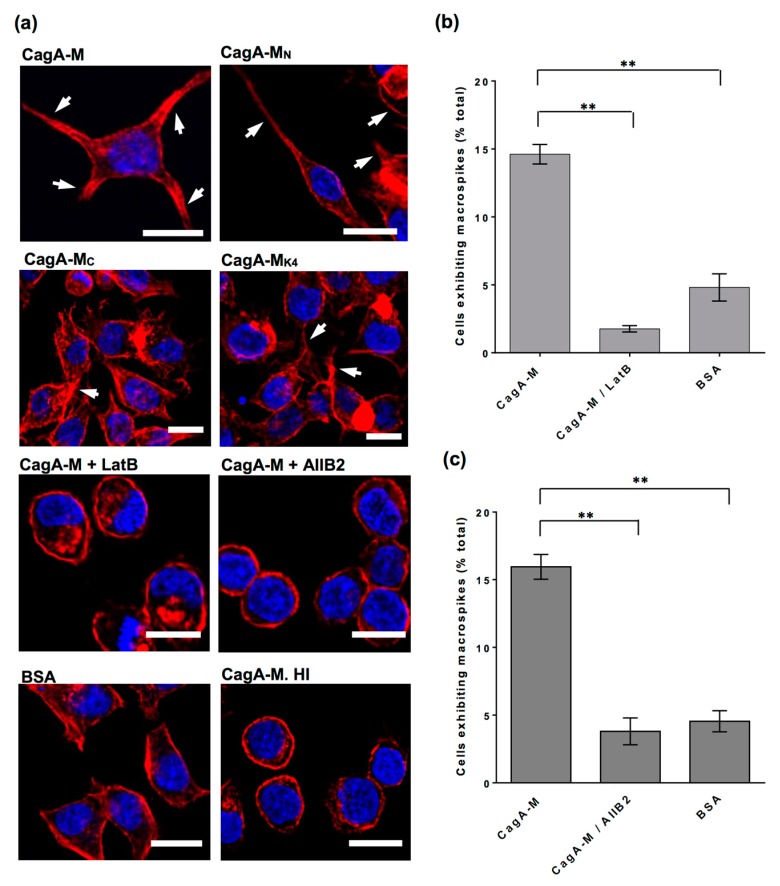
Confocal microscopy images showing AGS cells stained to detect actin cytoskeleton (red, Alexa Fluor 555-conjugated phalloidin) and the nucleus (blue, DAPI). (**a**) AGS cells were treated with various middle fragments of CagA, BSA or heat-inactivated CagA-M (CagA-M. HI) for 24 h. The macrospikes induced are indicated by white arrowheads. Scale bar, 10 μm. Each image is representative of approximately 150 cells examined in each sample; (**b**) AGS cells were treated with CagA-M with or without pre-treatment with latrunculin B (LatB). BSA-treated AGS cells served as a negative control for macrospike formation. Graph shows the percentage of AGS cells exhibiting macrospikes; (**c**) Graph showing the percentage of AGS cells exhibiting macrospikes. AGS cells were treated with CagA-M with or without pre-treatment with the specific β_1_ integrin function-blocking antibody (AIIB2). All data are mean values ± s.e.m. of three independent experiments (***, *p* < 0.001; **, *p* < 0.01). The data shown in panels (**b**,**c**) were determined from phase contrast microscopy images from independent experiments. A total of 350 cells per treatment condition evaluated.

Based on the morphogenic effect of CagA-M, we postulated that the middle fragment of CagA could trigger actin cytoskeletal rearrangement in the host cell, leading to altered cell morphology. To test this hypothesis, AGS cells exposed to BSA or CagA-M were stained with fluorophore-conjugated phalloidin, which labels polymerized actin, and inspected by confocal microscopy. Unlike BSA-treated cells, CagA-M-treated cells had major actin rearrangement with polymerized actin in high abundance at the macrospike protrusions (Figure 3a). Furthermore, when AGS cells were pre-treated with latrunculin B (LatB), a compound that inhibits actin polymerization and disrupts actin rearrangement [32,33], CagA-M-induced macrospike formation was virtually abolished (Figure 2a,c and Figure 3a,b). These results suggest that actin rearrangement contributes to CagA-M-induced altered cell morphology.

Human cell surface receptor integrins play crucial roles in regulating actin dynamics [34]. To determine whether β_1_ integrin activation is required for CagA-M-induced morphological change, AGS cells were pre-treated with the β_1_ integrin function-blocking antibody AIIB2. In contrast to pre-treatment with BSA (control), AIIB2 pre-treatment greatly reduced CagA-M-induced macrospike formation (Figure 2a,c and Figure 3a,c). These findings indicate that β_1_ integrin activity mediates the morphological changes induced by the CagA middle fragment.

### 2.3. Amino Acid Residues 257 to 266 and the Lipid-Binding Site within the Middle Fragment of CagA Are Essential for Induction of Host Cell Morphological Changes

To identify the protein elements that play a role in CagA-M-induced cell morphological changes, the morphogenic effects of variant proteins CagA-M_N_, CagA-M_C_, and CagA-M_K4_ were examined in parallel with CagA-M. CagA-M_N_ (aa 257–807), which has the same N-terminus as CagA-M but lacks 73 amino acid residues at the C-terminus (Figure 1), was also capable of stimulating macrospike formation in the AGS cells (Figure 2 and Figure 3a). In contrast, the proteolytically stable core fragment CagA-M_C_ (267–807), which also lacks 10 amino acids at the N-terminus in addition to the aforementioned C-terminus truncation (Figure 1), showed a significantly reduced ability to stimulate morphological changes (Figure 2 and Figure 3a). Minimal morphological change was similarly observed in AGS cells treated with CagA-M_K4_ (257–880; K613A/K614A/K617A/K621A) (Figure 2 and Figure 3a), a variant of CagA-M that harbors lysine-to-alanine substitutions at four positions in the putative PS-binding region (Figure 1). Taken together, these observations indicate that the N-terminal 10 amino acid residues (257–266) and the PS-binding region (residues 613, 614, 617, and 621) in the middle domain of CagA play crucial roles in stimulating actin rearrangement and morphological changes in AGS cells.

### 2.4. The Middle Fragment of CagA Immobilized on Latex Beads Is Capable of Binding and Entering AGS Cells

Our data suggested that altered AGS cell morphology induced by CagA-M or CagA-M_N_ was triggered by activation of β_1_ integrin signaling and actin-driven formation of membrane protrusions, both arising from direct interactions of the protein molecules with the AGS cells. These signaling responses are reminiscent of those involved in ligand-induced endocytosis; a process that is physiologically used for ingestion of nutrients, and that is commonly ‘hijacked’ by bacteria and viruses to gain entry into eukaryotic cells. We, therefore, hypothesized that the middle fragment of CagA alone is sufficient for triggering its own uptake into the host cell by activating the host machinery for endocytosis, including actin-driven formation of protrusions (macrospikes), through activation of β_1_ integrin. 

**Figure 4:**
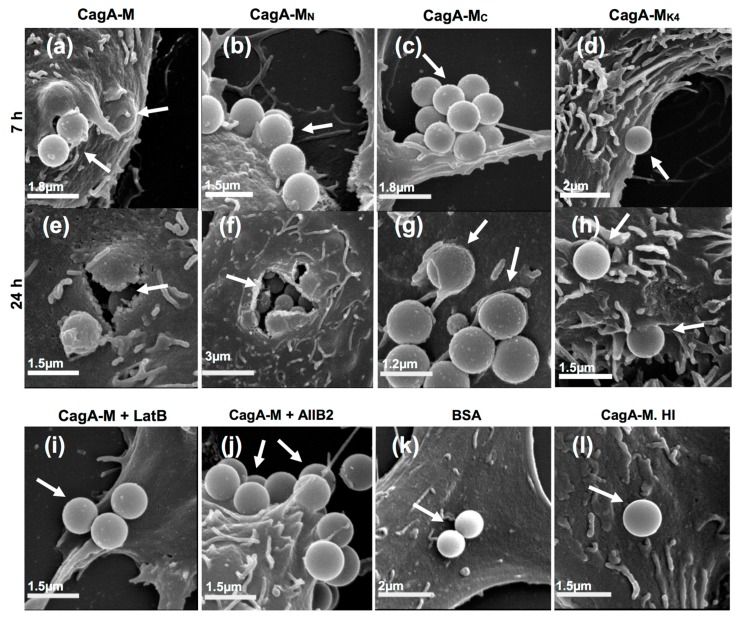
Scanning electron microscopy of AGS cells incubated with 1.1 μm latex beads coated with CagA-M or one of its variants. Cells were incubated for seven or 24 h with latex beads pre-coated with CagA-M, CagA-M_C_, CagA-M_N_, or Cag-M_K4_ (**a**–**h**). The micrographs in the bottom row show AGS cells that were pre-treated with either LatB or AIIB2 prior to 24-h incubation with CagA-M-coated beads; AGS cells incubated for 24 h with latex beads coated with BSA or latex beads coated with heat-inactivated CagA-M (CagA-M. HI) served as negative controls. Scale bars are as shown.

**Figure 5:**
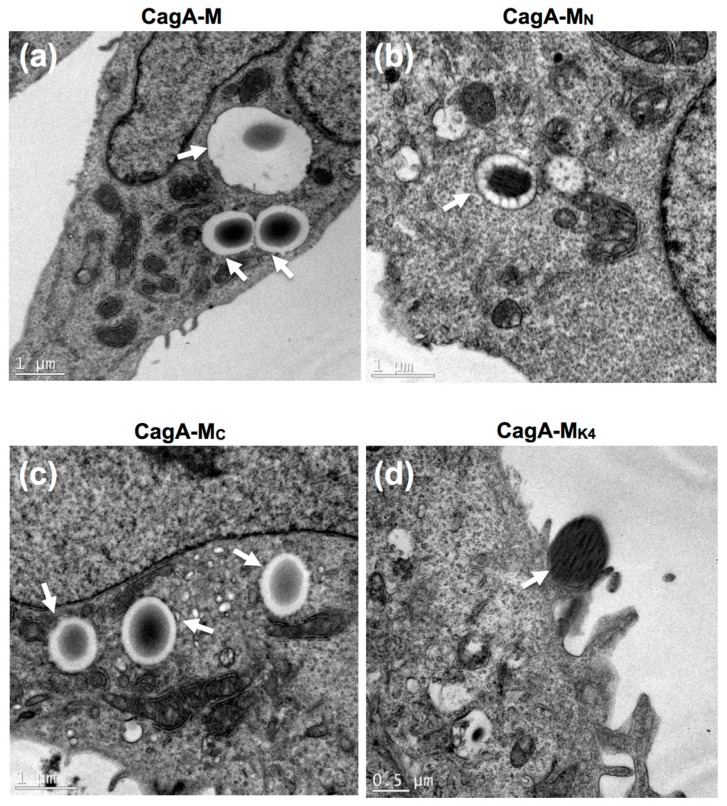
Transmission electron micrographs showing cross-sections of AGS cells incubated for 24 h with 1.1 μm latex beads pre-coated with CagA-M, CagA-M_C_, CagA-M_N_, or CagA-M_K4_ (bead/cell ratio 50:1). White arrowheads show internalized beads (**a**–**c**) or a bead found partially enveloped by a cell surface structure that resembles an endocytic cup (**d**). Scale bars are as shown.

To test this hypothesis, we employed latex beads (1.1 µm in diameter) that are in the same size range as most bacteria. Such latex beads have been widely used for visualizing the process of ligand-induced endocytosis at the ultrastructural level. Latex beads coated with CagA-M, CagA-M_N_, CagA-M_C_, CagA-M_K4_, BSA (negative control) or heat-inactivated CagA-M (negative control) were incubated with AGS cell monolayers for up to 24 h. Scanning electron microscopy (SEM) revealed that after 7 h and 24 h incubation, CagA-M- and CagA-M_N_-coated beads were readily detectable on the surface of AGS cells (Figure 4 and Table 1). Moreover, after 7 h incubation, lamellipodia were evident around the majority of the bound CagA-M- and CagA-M_N_-coated beads, forming so-called endocytic cups beneath the beads (Figure 4a,b). Actin-rich endocytic cups are often observed during actin-driven endocytic processes [35]. Strikingly, following 24 h incubation, gastric cell-internalized beads coated with CagA-M or CagA-M_N_ were clearly visible at sites where the AGS cell membrane had ruptured during the sample preparation (Figure 4e,f). In addition, numerous round “bumps” (convex membrane deformations) pointing to the locations of further, hidden internalized beads were observed on the AGS cells incubated with CagA-M- and CagA-M_N_-coated beads (Figure 4a,e,f). Internalization of CagA-M- and CagA-M_N_-coated beads into the AGS cells was confirmed by transmission electron microscopy (TEM), which showed that the beads were located inside intracellular vesicles that resembled phagosomes and macropinosomes (Figure 5a,b). In contrast, latex beads coated with BSA or heat-inactivated CagA-M were only rarely found on the surface of the AGS cells (Figure 4k,l; Table 1), and never inside the cells (Table 1). These results suggest that both CagA-M and CagA-M_N_ are able to bind to AGS cells and subsequently gain entry into the host cell cytoplasm. Our data also indicate that a folded conformation of CagA-M is essential for the uptake.

We next examined whether residues 257–266 and the positively-charged PS-binding patch on the CagA surface, that were shown to be important for inducing morphological changes and actin cytoskeletal rearrangement in the AGS cells, are also required for mediating cell binding and internalization of CagA-M. Using a similar approach, we performed SEM and TEM on the AGS cells incubated with CagA-M_C_- or CagA-M_K4_-coated beads. Compared to CagA-M, beads coated with the core fragment CagA-M_C_ were only scarcely found on the AGS cell surface (Figure 4c). Moreover, endocytic cups or lamellipodia were only very rarely observed around the CagA-M_C_-coated beads (Figure 4g). Although TEM provided evidence of internalized CagA-M_C_-coated beads (Figure 5c), semi-quantitative analysis of their relative abundance indicated that CagA-M_C_ has a reduced capacity to enter AGS cells compared to CagA-M- and CagA-M_N_-coated beads (Table 1 and Table 2). CagA-M_K4_-coated beads were found in even lower abundance on AGS cells (Figure 4d, Table 1 and Table 2). The small proportion of cell-associated CagA-M_K4_-coated beads, minimal lamellipodia formation after 24-h incubation (Figure 4h and Figure 5d) and significantly reduced engulfment (Table 1; *p* < 0.0001 compared with CagA-M engulfment, one-way ANOVA) suggested that they can, at best, only weakly induce their own uptake into AGS.

Taken together, the SEM and TEM results provide novel evidence that the middle fragment of *H. pylori* CagA can trigger its own uptake into human gastric epithelial cells. Moreover, these findings suggest that amino acid residues 257–266 and the PS-binding site (residues 613, 614, 617, and 621) play important roles in the interaction of the CagA middle fragment with AGS cells and its subsequent entry into the host cells. 

### 2.5. Actin Cytoskeletal Rearragnement and β_1_ Integrin Activation Are Essential for the Uptake of CagA Middle Fragment-Coated Beads into AGS Cells

We next examined whether activation of β_1_ integrin and/or actin cytoskeletal rearrangement were required for the middle fragment of CagA to trigger its own uptake into the host cells. To test this hypothesis, we analyzed the interaction of CagA-M-coated beads with AGS cells in the presence and absence of LatB or the β_1_ integrin function-blocking antibody AIIB2. SEM (Figure 4i and Table 1) and TEM (data not shown) analysis of LatB-pre-treated AGS cells showed beads on the cell surface but not inside the cells, indicating that LatB blocked endocytosis of CagA-M-coated beads. These observations suggest that uptake of CagA-M-coated beads into AGS cells strictly requires actin polymerization and actin rearrangement. Similarly, pre-treatment of AGS cells with AIIB2 dramatically inhibited endocytosis of CagA-M-coated beads as suggested by SEM (Figure 4j and Table 1) and TEM analyses (data not shown). These findings are in line with the hypothesis that entry of CagA-M-coated beads into AGS cells relies on both actin polymerization/rearrangement and activation of β_1_ integrin. 

## 3. Discussion

CagA is a multifunctional virulence factor of *H. pylori*. Owing to its oncogenic and proinflammatory potential, the molecular events that follow CagA translocation into host cells have been thoroughly investigated and are well understood. However, little is known about the mechanisms by which CagA is translocated into the host cell and some key questions remain unanswered. For example, is CagA ‘injected’ by the *H. pylori* T4SS directly into the host cell cytoplasm? Or, is it first secreted into the extracellular milieu and then taken up into the host cell by host-mediated processes? Meanwhile, binding of CagL, a structural subunit of the *H. pylori* T4SS, to the human transmembrane receptor α_5_β_1_ integrin has been shown to be essential for CagA translocation. Specific binding of CagA itself to β_1_ integrin is thought to be a prerequisite for CagA internalization into the gastric epithelial cells. And indeed, recently, amino acid residues required for β_1_ integrin binding have been identified in the middle region of CagA [28]. This new finding raises the possibility that the middle region of CagA could be responsible for mediating binding of secreted CagA to the host cell surface and, perhaps, even entry into the host cells via interaction with β_1_ integrin. 

The results of this study provide unprecedented evidence that the recombinant middle fragment of CagA comprising aa residues 257–807 is capable of binding to, and triggering its own uptake into, the host cell. Although we were unable to directly detect CagA-M or any of its variants inside AGS cells by immunostaining, the use of latex beads as microscopic markers enabled us to visualize the uptake process that took place upon interaction between AGS cells and different variants of the middle fragment of CagA. This approach revealed the formation of endocytic cups around cell-bound CagA-M- or CagA-M_N_-coated beads, suggesting that uptake of these CagA fragments likely involves the host endocytic machinery. Additionally, using highly-specific inhibitors, we demonstrated that the cell-binding and “invasive” properties of the middle fragment of CagA are strictly dependent on β_1_ integrin activity and actin dynamics. These findings are also in good agreement with the published data showing strong binding of a very similar CagA fragment (100 kDa, from aa 1 to approximately 885) to integrin α_5_β_1_ [23].

The cellular responses observed in association with the latex-bead mediated uptake of CagA-M or CagA-M_N_ are reminiscent of some of those described for the cellular uptake of chitosan-based nanospheres [36,37]. Chitosan is a natural glycan polymer widely used for intracellular delivery of macromolecules. Published data suggest that chitosan nanospheres enter mammalian cells generally via endocytosis, with the particular endocytic sub-pathways involved being determined by the surface chemistry of the nanospheres [36,37]. The fact that similar cell morphologies/responses were observed between the uptake of chitosan microspheres and the uptake of CagA-M-coated beads is in line with our observation that the latter also involved endocytosis. Despite this similarity, multiple lines of evidence in this study (Figure 4 and Figure 5, Table 1) indicate that the uptake of CagA-M-coated beads is highly specific. First, latex beads coated with CagA-M were readily taken up by AGS cells whereas no uptake of heat-inactivated CagA-M could be detected, indicating that the native conformation of CagA-M is essential for triggering the uptake. Second, uptake of CagA-M-coated beads was subject to inhibition by actin polymerization inhibitor or integrin α_5_β_1_ function-blocking antibodies, indicating that the uptake process requires actin polymerization and integrin α_5_β_1_ activity. Third, the reduced extent in the uptake of CagA-Mc- and CagA-MK4-coated beads compared to the uptake of CagA-M-coated beads shows that the uptake of CagA-M-coated beads specifically requires amino acid residues 257–266 and the phosphatidylserine-binding site in the CagA middle fragment. 

The observations that the middle fragment of CagA could induce development of macrospikes and a star-like morphology in AGS cells are equally noteworthy, although the pathophysiological significance of such phenotypic manifestations remains questionable. However, that CagA-M triggers macrospike formation or star morphology provides further evidence for the ability of the CagA middle region to interact with the host cell and subsequently trigger host cell cytoskeletal rearrangement; these responses are likely to contribute to the endocytic uptake of the middle fragment of CagA. Moreover, induction of the star morphology by CagA-M also required β_1_ integrin activity and actin polymerization/rearrangement, further confirming the importance of β_1_ integrin and actin in mediating CagA-host cell interaction.

The findings in this study also provide new insights into the structure-function relationship of CagA, which has a number of disordered regions (Figure 1), the role and significance of which is yet to be established. This study demonstrates that structurally disordered residues 257–266, which are located at the N-terminus of CagA-M, and form part of the linker between Domains I and II in the full-length CagA molecule, play an essential role in triggering actin rearrangement and morphological changes in AGS cells. Interestingly, our data show that this region is also important for inducing the internalization of the CagA middle fragment by host cells. In contrast, the disordered region comprising residues 808–880 at the C-terminus of CagA-M was not essential for the fragment’s cell binding and invasive properties. Given the large proportion of charged residues in the region aa 257–266 (E^257^ARDLLDERG^266^), it is tempting to speculate that these charged residues could be important for the interaction of CagA with the host cell and the subsequent actin rearrangement and endocytosis. Moreover, residues 303–404 within the middle fragment of CagA have previously been shown to bind integrin β_1_ in a yeast two hybrid screen [23] and could, therefore, be important for the activation of actin rearrangement and CagA uptake. It would be of interest to test these hypotheses in follow-up mutagenesis studies.

This study shows, for the first time, that structurally-disordered regions of CagA other than the EPIYA motifs-containing C-terminal region of CagA (~aa 890–1186, strain 26695 numbering) can also induce actin cytoskeletal rearrangement and phenotypic responses in the host cell. In particular, the results of this study pinpoint the important roles of β_1_ integrin receptor, actin polymerization/rearrangement and host phospholipid in CagA-host cell interactions and CagA host cell entry. Interestingly, translocation of full-length CagA by *H. pylori* into the host cell has previously been shown to require a similar set of host factors [21,24]. However, while the microscopy data in this study suggest that ligand-induced endocytosis is likely involved in the uptake of CagA-M or CagA-M_N_, the translocation of full-length CagA by *H. pylori* into AGS cells could not be attributed to any known endocytic mechanism in a previous study [21]. We cannot eliminate the possibility that the T4SS-independent uptake of CagA-M observed in this study occurs via a mechanism slightly different to that of the T4SS-mediated translocation of full-length CagA during *H. pylori* infection. Alternatively, it is possible that CagA-M and full-length CagA enter host cells via the same, albeit currently unknown, endocytic pathway and that only full-length CagA possesses the additional structural determinants that enable it to escape from the endocytic compartment into the host cell cytoplasm. In support of the notion that some T4SS effectors might be able to enter host cell cytoplasm via endocytosis, very recent findings show that VirE2, an effector protein of the *A. tumefaciens* T4SS, not only hijacks the clathrin-based endocytosis pathway to enter host cells, but also subsequently escapes from early endosomes [38]. Thus, further characterization of the interaction of VirE2, CagA and/or their respective cognate T4SSs with the host plasma membrane and endocytic machinery could provide new insights into understanding the inner workings of the enigmatic bacterial type IV secretion process.

We hypothesize that the auto-uptake mechanism supported by the findings in this study is one of the many steps involved in the complex process by which CagA is translocated into the host cell cytosol during *H. pylori* infection. It is plausible that during infection, upon contact of the T4SS with the host cell, CagA is first secreted by the T4SS to the surface of the bacterium, followed by integrin-binding and, hence, auto-uptake via endocytosis into the host cell. This model is in line with previous reports of CagA being detectable on the surface of *H. pylori* [21,39] and that the CagA fragment aa 1-613 is able to bind to integrin β_1_ extracellular domain [23]. While CagA can induce its own uptake into the host cell, the involvement of T4SS for CagA translocation might be required during *H. pylori* infection to ensure that CagA is secreted at specific regions of the host cell membrane (e.g., lipid rafts). The latter could be achieved via the interactions of CagL, and other structural components of T4SS, such as CagI and CagY, with host cell integrin receptors [23,24]. By providing evidence for the auto-uptake of the CagA middle fragment into the host cell, this present work may open new avenues of research to better understand the molecular mechanism of the type IV secretion process.

## 4. Materials and Methods

### 4.1. Gene Synthesis, Cloning, Protein Expression, and Purification

The coding sequences for *H. pylori* ATCC 26695 (Genbank ID AAD07614) CagA fragments CagA-M_N_ (aa 257–807) and CagA-M_K4_ (aa 257–880; K613A/K614A/K617A/K621A) were synthesized and cloned into vector pET151/D-TOPO (Genscript, Piscataway, NJ, USA), resulting in expression vectors with N-terminal His_6_ tag and tobacco etch virus (TEV) protease cleavage site-containing linker (GKPIPNPLLGLDSTENLYFQ↓GIDPFT). For protein expression, *E. coli* strain BL21 DE3 (Merck, Darmstadt, Germany) was transformed by the plasmids containing CagA fragments CagA-M_N_, CagA-M_K4_, and previously described CagA-M (aa 257–880) and CagA-M_C_ (aa 267–807) [30]. All CagA fragments were expressed and purified following the previously published procedure [30], and contain the amino acid sequence GIDPFT at their N-terminus as a cloning artefact. Protein concentration was determined using the Bradford method [40] and protein purity was estimated to be at least 90% by SDS-PAGE (Supplementary Figure S1). The expression levels and purity of variants CagA-M_N_, CagA-M_K4_ were very similar to those of CagA-M and CagA-M_C_ (Supplementary Figure S1), suggesting that the substitutions did not affect the integrity or stability of the protein fold. Screening for conditions that promote stability and solubility of the prepared fragments using aggregation spin test identified 30 mM sodium acetate pH 4.6, 200 mM NaCl as the optimum buffer.

### 4.2. Tissue Culture and Treatment with CagA Fragments

The human gastric adenocarcinoma cell line AGS (ATCC CRL-1739) was routinely maintained in Roswell Park Memorial Institute (RPMI) tissue culture medium (Gibco, Gaithersburg, MD, USA) containing 10% (*v*/*v*) heat-inactivated fetal bovine serum as described previously [41]. For treatment with CagA fragments, glass coverslips (12 mm diameter) in 24-well polystyrene tissue culture plates (BD Biosciences, Franklin Lakes, NJ, USA) were seeded with 0.7 × 10^5^ AGS cells and incubated overnight at 37 °C with 5% CO_2_ to obtain a final culture confluency of 30–50%. Following washing with phosphate-buffered saline (PBS) pH 7.4, a 330 µL aliquot of serum-free RPMI containing CagA-M, CagA-M_N_, CagA-M_C_, or CagA-M_K4_ (1 mg/mL in 100 mM NaH_2_PO_4_-Na_2_HPO_4_ pH 7.4, 150 mM NaCl) was added to each well and plates were incubated at 37 °C in 5% CO_2_. BSA and heat-inactivated CagA-M (denatured at 95 °C for 45 min) were used as negative controls. After 7 h- or 24 h-incubation, the coverslips were washed gently with PBS buffer before fixation with 3.8% (*w*/*v*) paraformaldehyde (PFA) in PBS at room temperature for 15 min. AGS cell morphology was observed using an inverted bright field microscope CXK41 (Olympus, Tokyo, Japan) for phase contrast analysis equipped with a Nikon digital sight DS-Fi1 camera and Nikon imaging software for image capture. Lengths of macrospikes were determined from the phase contrast micrographs using the Analyze function in ImageJ (version 1.48). AGS cells whose average macrospike length was in excess of 4 µm were considered macrospike-exhibiting cells. Macrospike formation was observed at both 0.5 mg/mL and 1 mg/mL concentrations of CagA-M. However, because macrospike formation triggered by 1 mg/mL CagA-M was the most reproducible between independent experiments, detailed quantitative and qualitative analyses, therefore, focussed on treatments performed at 1 mg/mL protein concentration.

### 4.3. Inhibition of Actin Cytoskeleton Polymerization and β_1_ Integrin Activity

LatB was used to inhibit actin polymerization in AGS cells (Sapphire Biosciences, Redfern, New South Wales, NSW, Australia). LatB disrupts microfilament organization by binding G-actin monomers and preventing the F-actin assembly [32,33]. Activity of β_1_-containing integrins on the cell surface was inhibited using the β_1_ integrin-specific blocking monoclonal antibody, AIIB2 (obtained from the Developmental Studies Hybridoma Bank; the antibody was created by the National Institute of Health (NIH) and maintained at the University of Iowa, Department of Biology, Iowa City, IA, USA). 

To perform the inhibition assays, AGS cells on 12-mm glass coverslips were pretreated at 37 °C, 5% CO_2_ with 0.5 µM LatB for 2 h, or 2 µg/mL rat anti-integrin β_1_ (AIIB2) antibody for 1 h. Purified CagA-M (1 mg/mL) was then added to the cells and incubation continued for another 24 h. The cells were then fixed with 4% (*w*/*v*) PFA in PBS for 15 min at room temperature, after which they were washed twice with 500 µL cold PBS. Cell morphology was then examined by phase contrast bright field microscopy as described above. Samples were then stained with Alexa Fluor 555-conjugated Phalloidin (ThermoFisher Scientific, Waltham, MA, USA) (see section on “Phallodin staining and fluorescence microscopy” for details) to assess the effect of the inhibitors on the AGS cell morphology. 

### 4.4. Latex Bead Binding Assay

To coat latex beads with CagA-M or its variants, approximately 5 × 10^6^ latex beads (1.1 µm diameter, Sigma) were washed three times with 400 μL of coupling buffer (100 mM NaPi pH 7.4, 150 mM NaCl) and mixed with 3 mg/mL of CagA-M, CagA-M_N_, CagA-M_C_, CagA-M_K4_, BSA (fatty acid free, Sigma) or heat-inactivated CagA-M (CagA-M. HI) in 100 mM NaH_2_PO_4_-Na_2_HPO_4_ pH 7.4, 150 mM NaCl. The amount of protein in each case was in excess of that required to treat an AGS monolayer using soluble protein. Following overnight incubation at 4 °C, the bead-protein suspension was incubated at 37 °C for 1 h, then washed with coupling buffer, sonicated at the lowest output power (three seconds, three times), and centrifuged at 7000× *g* for 5 min. Bradford assay demonstrated that depletion of protein from solution was consistent across all tested fragments. The bead-protein pellet was resuspended in blocking buffer (100 mM NaH_2_PO_4_-Na_2_HPO_4_ pH 7.4, 150 mM NaCl, 2% (*w*/*v*) BSA) to block any non-specific sites, and incubated at 37 °C for 1 h. Samples were then centrifuged and the pellet was washed with Dulbecco’s phosphate-buffered saline (DPBS) containing 1 mg/mL BSA. Protein-coated bead suspension (10 µL in 320 µL DPBS containing 0.2 mg/mL BSA) was added to AGS monolayers at a final bead to cell ratio of 50:1 and incubated for seven or 24 h at 37 °C with 5% CO_2_. AGS cells were then washed twice with 1 mL warm RPMI media to remove unbound or loosely bound beads, followed by fixation with 2.5% *v*/*v* glutaraldehyde for 2–3 h at room temperature for SEM and TEM or with 3.8% (*w*/*v*) PFA for phase contrast microscopy. AGS cells treated with BSA-coated beads (bead-only control) exhibited normal cell morphology similar to that of untreated AGS cells up to at least 24 h post-treatment.

### 4.5. Staining of Actin Microfilaments and Confocal Laser Scanning Microscopy

To stain actin microfilaments in AGS cells, specimens on glass coverslips were initially blocked with 1% *w*/*v* fatty acid-free BSA in PBS for 1 h and then permeabilized with 0.1% (*v*/*v*) Triton X-100 at room temperature. Actin cytoskeleton was fluorescently labelled with the Alexa Fluor 555-conjugated Phalloidin (ThermoFisher Scientific, Waltham, MA, USA). Cell nuclei were visualized using conventional DAPI (4′,6-diamidino-2-phenylindole) staining. Following staining, coverslips were mounted in Mowiol. Images were acquired using a confocal laser scanning microscope (Olympus, FV500 Fluorescence Life Time Imaging Microscopy, Australia) fitted with a Pico Star CCD camera and FluoView software for image capture, were false-coloured, overlaid, and scale bars added using Image J software [42]. The contrast of images was globally optimized by applying the Auto Contrast function in Adobe Photoshop Elements software (v.12) to the entire image of each individual image file.

### 4.6. Scanning Electron Microscopy (SEM)

Specimens on coverslips were fixed in 2.5% (*v*/*v*) glutaraldehyde in 0.1 M sodium cacodylate (pH 7.3) for 2–3 h at room temperature and then treated with 1% (*w*/*v*) osmium tetroxide (OsO_4_) for 1 h, after which they were dehydrated in a graded series of ethanol (50–100% [*v*/*v*]) and then subjected to critical-point drying with hexamethyldisilazane (HDMS). Samples were then fixed on ultra-pre-cleaned copper grids, sealed with a thin layer of carbon glue to increase conductivity, covered with a 10-nm thick gold film and examined using a Hitachi S-570 scanning electron microscope with an acceleration voltage of 15–25 V and a working distance of 6 mm. The sample images were processed and visualized using Spectrum software. Semi-quantitative analysis of the number of intracellular beads per sample was carried out by averaging the total count of intracellular beads in 20 different microscopy fields to yield the mean number of intracellular beads per field of view (Table 2). On average, a total of 60 cells per sample were analyzed.

### 4.7. Transmission Electron Microscopy (TEM)

Specimens on coverslip were fixed with 2.5% (*v*/*v*) glutaraldehyde in 0.1 M sodium cacodylate (pH 7.3) for 3 h at room temperature and then washed with 0.1 M sodium cacodylate (pH 7.3), after which cell monolayers were scraped and transferred into sterile 1.7-mL microcentrifuge tubes. Following treatment with 1% (*v*/*v*) OsO_4_ and centrifugation (94× *g*, 5 min), cell pellets were serially dehydrated with ethanol (50–100% [*v*/*v*]). Cell pellets were then dried with serial ratios of absolute (100%) ethanol-HDMS mixture (2:1, 1:1, 1:2) followed by immersing in 100% HDMS for 40 min. Cell pellets were subsequently embedded in Epon Epoxy resin and incubated at 60 °C for 48 h. Samples were sectioned by ultramicrotomy, placed on copper grids, and analysed using a Hitachi-H7500 transmission electron microscope equipped with a digital camera (Gatan). The contrast of images was adjusted globally using the graphic software CorelDraw Graphics Suite version X7 (Corel Inc., Austin, Texas, TX, USA, 2014). Semi-quantitative analysis of the number of intracellular beads per sample was carried out by averaging the total count of intracellular beads in 20 different microscopy fields to yield the mean number of intracellular beads per field of view (Table 2). On average, a total of 60 cells per sample were analyzed.

## Figures and Tables

**Table 1:** The number of extracellular and engulfed latex beads coated with various CagA middle fragments determined from the SEM images of treated AGS cells.

Treatment	Average Number of Beads Per Field ^a^ (Mean ± S.D.)	
	Engulfed Beads ^b^	Extracellular Beads
CagA-M	4.1 ± 1.4	4.2 ± 1.1
CagA-M_N_	4.5 ± 1.7	4.9 ± 1.5
CagA-M_C_	2.4 ± 1.1	2.5 ± 1.2
CagA-M_K4_	1.2 ± 0.4	1.8 ± 0.7
BSA	0 ± 0	0.2 ± 0.4
CagA-M. HI	0 ± 0	0.2 ± 0.5
CagA-M + LatB	0 ± 0	0.2 ± 0.4
CagA-M + AIIB2	0 ± 0	0.2 ± 0.5

Remarks: ^a^ A total of 20 SEM fields of view were analyzed per treatment condition. ^b^ The numbers are likely to be underestimates of the actual number of engulfed beads because of the nature of the SEM technique. Only engulfed beads that were clearly visible as a result of membrane rupture or round bumps on the cell membrane that are the size of a latex bead (1.1 µm in diameter) were counted. Intracellular beads that were not visible or could not be unambiguously identified were not counted.

**Table 2:** Extent of internalization of latex beads coated with CagA middle fragment variants by AGS cells.

	CagA-M	CagA-M_N_	CagA-M_C_	CagA-M_K4_
Average number of beads per field	3.2	3.4	2.2	1.1

## Supplementary Materials

The following are available online at www.mdpi.com/2072-6651/9/8/237/s1. Figure S1: Coomassie Blue-stained 12% SDS–PAGE gels of the recombinant CagA fragments used in this study.
