# Supplementary material for: The Middle Fragment of Helicobacter pylori CagA Induces Actin Rearrangement and Triggers Its Own Uptake into Gastric Epithelial Cells
Source: Toxins (Basel). 2017 Jul 28;9(8):237. doi: 10.3390/toxins9080237 (PMC5577571; doi:10.3390/toxins9080237)
Supplement: Supplementary file 1 [file toxins-09-00237-s001.pdf]

Supplementary Materials: The Middle Fragment of *Helicobacter pylori* CagA Induces Actin Rearrangement and Triggers Its Own Uptake into Gastric Epithelial Cells

Abolghasem Tohidpour [1], Rebecca J. Gorrell[1,2], Anna Roujeinikova [1,2,*] and Terry Kwok [1,2,*]

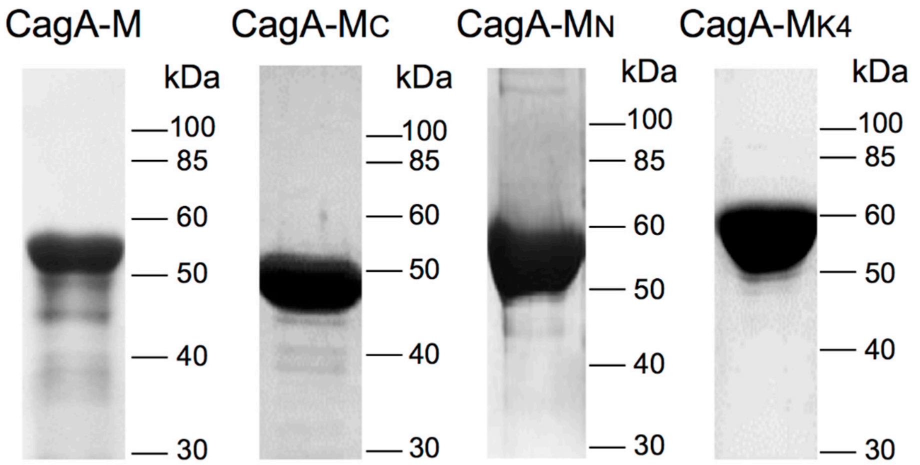

Figure S1. Coomassie Blue-stained 12% SDS–PAGE gels of the recombinant CagA fragments used in this study. Molecular weight markers (kDa) are as indicated on the right of each panel. Lane 1, CagA-M (a.a 257-880); Lane 2, CagA-MC (a.a 267-807); Lane 3, CagA-MN (a.a 257-807); Lane 4, CagA-MK4 (a.a 257-880).
